# Case report: Excessive daytime sleepiness as a presenting manifestation of autoimmune glial fibrillary acidic protein astrocytopathy

**DOI:** 10.3389/fimmu.2023.1302514

**Published:** 2023-12-20

**Authors:** Mingyang Tang, Shuo Huang, Weitong Guo, Junfang Zhou, Zhencan Huang, Wanru Li, Qingqing Sun, Zan Wang

**Affiliations:** Department of Neurology, The First Hospital of Jilin University, Changchun, Jilin, China

**Keywords:** case report, autoimmune glial fibrillary acidic protein astrocytopathy, glial fibrillary acidic protein, narcolepsy, sleep apnea-hypopnea syndrome

## Abstract

Autoimmune glial fibrillary acidic protein astrocytopathy (GFAP-A) is a recently discovered autoimmune inflammatory disease of the central nervous system. It presents with a variety of clinical symptoms, including fever, seizures, psychiatric symptoms, limber weakness, and sensory symptoms. However, the symptoms of sleep disorders have not been sufficiently addressed. Here, we report a case of GFAP-A in which the patient complained of excessive daytime sleepiness and an excessive need for sleep. Our patient was a 58-year-old male who experienced excessive daytime sleepiness for 50 days following SARS-CoV-2 infection. He was diagnosed with coronavirus disease 2019 on June 1st. On the 7th of June, he experienced excessive daytime sleepiness, nausea, reduced food intake, lower limb weakness, and dysuria. Subsequently, his sleepiness significantly deteriorated on July 21st. Five months prior, the patient underwent laparoscopic partial right nephrectomy for clear-cell renal cell carcinoma. Brain MRI revealed abnormal hyperintense lesions in the pontine brain and around the mesencephalic aqueduct on T2 and T2-fluid attenuated inversion recovery (T2-FLAIR) sequences However, these lesions did not exhibit any pathological enhancement. Spinal cord MRI revealed lesions in the C6–C7 and T2–T3 segments on the T2 sequence. His Epworth Sleepiness Scale (ESS) score was 16 (reference range, <10), and 24-hour polysomnography supported the diagnosis of rapid-eye-movement sleep disorder and severe sleep apnea-hypopnea syndrome. Glial fibrillary acidic protein IgG antibodies were detected in the cerebrospinal fluid (1:32, cell-based assay) but not in the serum. The level of hypocretin in the cerebrospinal fluid was 29.92 pg/mL (reference range ≥110 pg/mL), suggesting narcolepsy type 1. After treatment with corticosteroids for approximately 1 month, the patient showed considerable clinical and radiological improvement, as well as an increase in hypocretin levels. Although repeated polysomnography and multiple sleep latency tests suggested narcolepsy, his ESS score decreased to 8. Our findings broaden the range of clinical manifestations associated with GFAP-A, thereby enhancing diagnostic and therapeutic strategies for this disease. Additionally, our results indicate a potential common autoimmune mechanism involving GFAP-A and orexin system dysregulation, warranting further investigation.

## 1 Introduction

Autoimmune glial fibrillary acidic protein astrocytopathy (GFAP-A) is a corticosteroid-responsive novel autoimmune inflammatory central nervous system disease defined by Fang (1) et al. in 2016; it usually presents as meningitis, encephalitis, myelitis, optic neuritis, or a combination of the above. The characteristic MRI features of GFAP-A are linear radially enhancing lesions perpendicular to the lateral ventricles, meningeal enhancement, and early brainstem and spinal cord involvement (2). Additionally, a small proportion of patients develop reversible lesions in the splenium of the corpus callosum (3, 4). The detection of glial fibrillary acidic protein (GFAP)- IgG in the cerebrospinal fluid (CSF) by both tissue- and cell-based testing serves as a biomarker for GFAP-A (5).

Most GFAP-A patients present with inflammatory CSF with elevated levels of CSF proteins and lymphocyte-predominant pleocytosis, which may last for months. Additionally, patients may show transiently increased CSF adenosine deaminase levels during the first month after disease onset (6, 7). Furthermore, these patients may have a history of infections, tumors, or other autoimmune illnesses such as thyroid dysfunction or type 1 diabetes. GFAP-A patients may also experience headache, fever, seizures, psychiatric symptoms, limb weakness, sensory symptoms, abnormal autonomic nervous function, or blurred vision (1, 6, 8, 9). In 2021, Gao et al. (10) reported a patient presenting with area postrema syndrome with intractable nausea and vomiting as the predominant symptoms. The MRI revealed abnormal hyperintensities on the T2 sequence in the dorsal medulla oblongata and right pontine arm.

Here, we report the case of a patient with excessive daytime sleepiness associated with GFAP-A, which has rarely been reported as a predominant symptom, to improve physicians’ understanding of GFAP-A.

## 2 Case description

**Figure 1:**
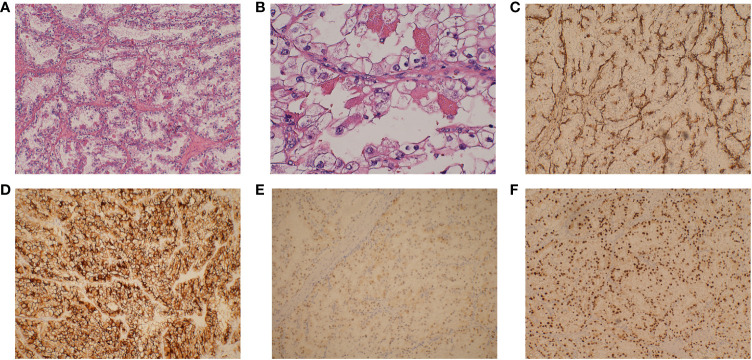
Pathological examination of this renal mass indicates the presence of clear cell renal cell carcinoma. **(A)** The tumor is arranged in vesicular or nested clusters, with numerous interstitial capillaries, a clear cytoplasm (original magnification ×100), and **(B)** conspicuous and eosinophilic nucleoli (original magnification ×400). **(C)** Tumor cells that are positive for vimentin (original magnification ×100). **(D)** Tumor cells that are positive for CD10 (original magnification ×100). **(E)** Tumor cells that are positive for PAX2 (original magnification ×100). **(F)** Tumor cells that are positive for PAX8 (original magnification ×100).

A 58-year-old male patient experienced excessive daytime sleepiness for 50 days after being infected with SARS-CoV-2. On June 1st, the patient presented with fever, reaching a maximum temperature of 38.5°C, and a cough, and was diagnosed with SARS-CoV-2 infection. Following treatment, his symptoms resolved. On June 7th, the patient exhibited excessive daytime sleepiness, slept for 12–13 hours daily, and experienced sensations of nausea, reduced food intake, weakness in the lower limbs, and dysuria. After the onset of sleepiness, the patient did not experience fever recurrence, and there was no significant weight loss. On July 21st, the patient’s symptoms markedly worsened. During this time, the patient slept for 20 h per day, even during meals and conversations. He fell asleep in seconds, showing no response to external stimuli and talking in his sleep, and awakened spontaneously after approximately 20 min, reporting nightmares during his recent sleep. The patient had undergone laparoscopic partial right nephrectomy for renal clear-cell carcinoma 5 months prior (**Figure 1**). However, he had not been diagnosed with any other illnesses and did not regularly use any drugs, toxins, or psychotropic substances. The patient did not report any previous subjective sleep complaints, noted that he slept for 6–7 hours daily before the onset of the disease, and did not exhibit cataplexy, nocturnal shouting, sleep paralysis, or hypnagogic hallucinations. The patient was 169 cm tall, weighed 70 kg, and had a body mass index of 24.5 kg/m^2^. A neurological examination upon admission revealed somnolence and weakness in both lower limbs (Medical Research Council Scale score of 4 +). The Epworth Sleepiness Scale (ESS) is the most commonly used self-reported questionnaire used by clinicians and researchers to measure daytime sleepiness. A score of ≥10 is indicative of abnormal sleepiness, with higher scores indicating greater severity of sleepiness (10). The patient attained a score of 16 on the ESS scale. Blood analysis showed that his tumor marker carbohydrate antigen 724 level was 22.90 U/mL (reference range <10 U/mL), while his thyroid function and levels of antineutrophil cytoplasmic antibodies, antinuclear antibodies, anti-dsDNA antibodies, anti-Jo-1 antibodies, anti-Sm antibodies, anti-PM-Scl antibodies, and anti-Ro52 antibodies were normal.

**Figure 2:**
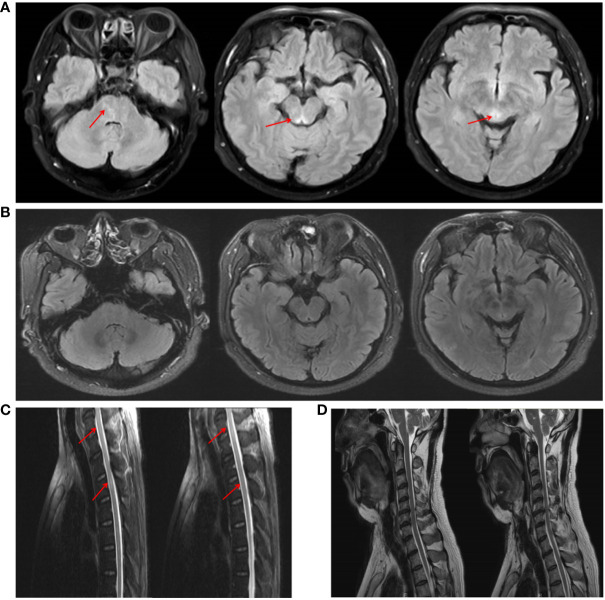
MRI imaging of the patient before and after treatment. **(A)** Brain MRI showed abnormal signals around the mesencephalic aqueduct and the pontine brain on the T2-FLAIR sequence. **(C)** Spinal cord MRI revealed spinal cord lesions in the C6–C7 and T2–T3 segments on the T2 sequence. **(B, D)** After treatment, the patient’s intracranial lesions had almost disappeared.

Brain MRI revealed abnormal hyperintense lesions in the pontine brain and around the mesencephalic aqueduct T2 and T2-fluid attenuated inversion recovery (T2-FLAIR) sequences; however, these lesions did not show pathological enhancement. Spinal cord MRI revealed lesions in the C6–C7 and T2–T3 segments on the T2 sequence (**Figures 2A, C**). Moreover, the electroencephalogram was normal, ruling out epilepsy. PET-CT of the whole body also showed no evidence of abnormal cancerous hypermetabolism. Furthermore, the normal electroneurography and electromyography results of the limbs also ruled out Lambert-Eaton syndrome, as well as weakness due to myogenic disease or peripheral nerve damage.

**Figure 3:**
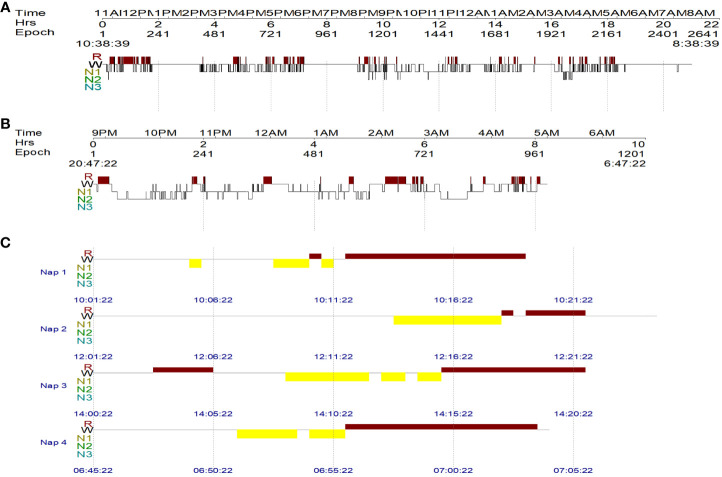
The patient’s sleep architecture before and after treatment. W = wakefulness; R = rapid eye movement (REM) sleep; N1, N2, and N3 = non-REM N1, N2, and N3 sleep stages. **(A)** The 24-hour PSG result of the patient before treatment, showing a total sleep time of 539.5 min. **(B)** Overnight PSG revealed a total sleep time of 393 min after treatment. **(C)** MSLT followed the overnight PSG and revealed 4 consecutive sleep-onset rapid eye movement periods.

The Multiple Sleep Latency Test (MSLT) is an objective measure for quantifying the level of daytime sleepiness by having the patient take a series of naps during the day. It requires the patient to take five 20-minute naps, two hours apart, during which the patient must remain awake. The first nap is usually scheduled 3 hours after the patient has awakened from the previous night’s polysomnography (PSG). Additionally, if a patient falls asleep and enters REM sleep within 15 min of sleep onset, it is recorded as a sleep-onset rapid eye movement period (SOREMP) (11). In this case, the patient’s sleepiness was too severe to perform MSLT; therefore, the patient underwent 24-hour PSG, which showed a total sleep time of 539.5 minutes (**Figure 3A**), sleep latency of 8.5 minutes, rapid-eye-movement sleep without atonia, and dream enactment behavior.

**Figure 4:**
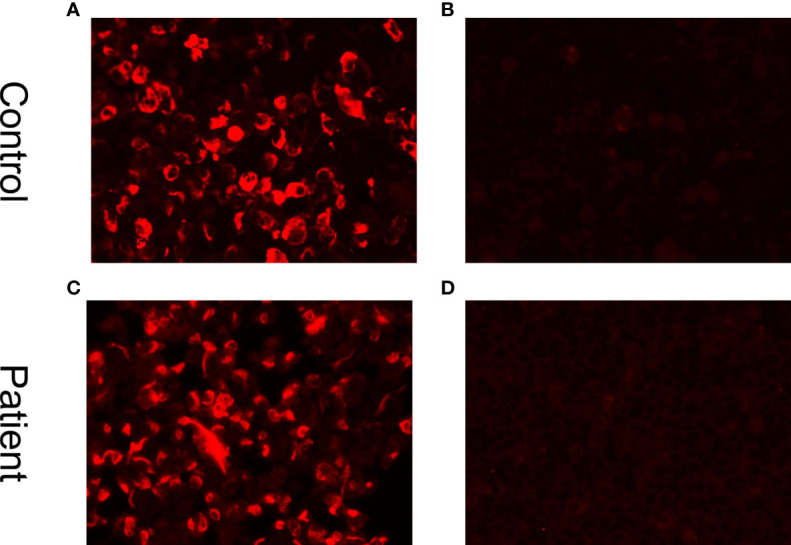
Cell-based indirect immunofluorescence assays demonstrate positivity for GFAP IgG antibodies. **(A)** Positive control with human GFAP IgG. **(B)** Negative control in a healthy individual. **(C)** Positive result in the CSF (titer at 1:32). **(D)** Negative result in serum.

The apnea-hypopnea index (AHI) is a measure of sleep apnea severity. The AHI assesses how often apnea (stopping breathing) and hypopnea (restricted and shallow breathing) events occur per hour during the night’s sleep. Typically, an AHI of less than five occurrences per hour is considered normal. In this case, the patient’s AHI was 75.6/h (obstructive AHI, 51.3/h; central AHI, 16.9/h; mixed AHI, 7.4/h), indicating Cheyne-Stokes respiration. Further examinations, including a CSF analysis on July 27, 50 days after the onset of symptoms, revealed an intracranial pressure of 115 mmH_2_O (reference range 80–180 mm H_2_O), a protein level of 1.44 g/L (0.15–0.45 g/L), and a leukocyte count of 140×10^6/L (reference range <8×10^6 cells/L), composed of 96% lymphocytes and 4% monocytes. Additionally, his glucose level was 1.98 mmol/L (reference range 2.3–4.1 mmol/L) and his IgG level was 151.93 mg/L (reference range <34 mg/L). The CSF analysis revealed the presence of oligoclonal bands in the CSF. Additionally, glial fibrillary acidic protein (GFAP) IgG antibodies were found to be positive in the CSF (1:32, cell-based assay) but not in the serum (**Figure 4**), whereas AQP4 antibodies and MOG antibodies were negative in both the CSF and serum. The level of hypocretin in the CSF was 29.92 pg/mL (reference range >200 pg/mL), and his detected human leukocyte antigen (HLA) allele was HLA DQB1*06:02/02:01. The patient was diagnosed with GFAP-A, narcolepsy type 1 (NT1), rapid eye movement sleep behavior disorder (RBD), and severe sleep apnea-hypopnea syndrome (SAHS), which included obstructive, central, and mixed apnea or hypopnea events, with central apnea manifesting as Cheyne-Stokes respiration (12).

The patient’s treatment included antiviral treatment and high-dose intravenous methylprednisolone at 1,000 mg/day for 3 days, followed by 500 mg/day for 3 days, 250 mg/day for 3 days, and 60 mg/day for 3 days, which was then switched to 60 mg/day orally. From the third day of steroid therapy, the patient’s sleepiness significantly reduced, and his sleep duration gradually decreased to daily levels. In the follow-up after one month of treatment, the patient’s sleepiness, weakness in both lower limbs, and dysuria resolved, and his ESS score dropped to 8. Follow-up CSF examination revealed decreased leukocyte (23×10^6/L), protein (0.96 g/L), and IgG levels (61.94 mg/L). The level of hypocretin in the CSF had increased to 87.81 pg/mL. Repeated PSG revealed a total sleep time of 393 min (**Figure 3B**), with a decrease in AHI to 26.4 times/h, and only obstructive respiratory events were observed. Despite these improvements, RBD persisted. The MSLT indicated a mean sleep latency of 6.3 min (normal >8 min) and the occurrence of four consecutive SOREMPs (**Figure 3C**). On repeat head MRI, the patient’s intracranial lesions had almost disappeared (**Figure 2B**). After 2 months of treatment, a follow-up examination showed that the patient’s spinal cord lesions had disappeared (**Figure 2D**).

## 3 Discussion

GFAP-A is a rare autoimmune inflammatory disease of the central nervous system, and its prevalence remains undefined. Among patients suspected of neurological autoimmune disease, Fang et al. (1) reported a GFAP-A prevalence of 0.134% (134 per 100,000) whereas Iorio et al. (13) reported a prevalence of 4.87% (22/451). In the population-based comparative research undertaken in Olmsted County, USA, the prevalence of GFAP-A was calculated to be 0.0006%(0.6/100,000) (14). In terms of patient characteristics, individuals with GFAP-A tend to have acute or subacute disease onset and can develop the disease at any age (median 44–50 years), with no significant difference in incidence between men and women (5, 8).

The etiology of GFAP-A remains unknown. Notably, 30–40% of GFAP-A patients have a history of infection, especially viral infections, before disease onset (8). Additionally, GFAP-A may be associated with neoplastic conditions. Fang et al. (1) reported that 38% of patients present with neoplasms, in contrast to the 34% (35/102) documented in the study of Flanagan et al. (5). Transitioning to specific neoplasm types, ovarian teratoma emerges as the most common, while other types are both rare and diverse. Remarkably, Flanagan et al. (5) previously reported a GFAP-A patient with combined renal cell carcinoma, which is consistent with our case.

GFAP is a type III intermediate filament protein expressed in mature astrocytes. It is an important component of the cytoskeleton and plays an essential role in the formation of the blood–brain barrier and myelination (15). The diagnosis of GFAP-A relies on the detection of GFAP antibodies in the CSF, currently considered a biomarker for this condition. However, the pathogenesis of GFAP-A remains elusive, with potential involvement of aberrant activation of the tumor necrosis factor pathway or GFAP peptide-specific CD8^+^ T lymphocytes, leading to astrocyte damage (16–18). Regardless, most patients with GFAP-A show inflammatory changes in the CSF, including increased white blood cell counts, elevated protein levels, and the presence of CSF-exclusive oligoclonal bands (8).

GFAP is predominantly expressed in the subpial and white matter, whereas in the gray matter, it is most abundant in the cerebral cortex and spinal cord, followed by the brainstem, hypothalamus, thalamus, hippocampus, amygdala, and basal ganglia (19). Moreover, the distribution of GFAP-A lesions on MRI is identical to that of histologically GFAP-enriched areas, often with linear radially enhanced lesions perpendicular to the lateral ventricles, meningeal enhancement, or early involvement of the brainstem and spinal cord (20).

There are some comparable clinical characteristics between our patient and previous reports. In this report, the middle-aged male patient had a SARS-CoV-2 infection prior to the onset of the disease and a history of neoplasia. Imaging studies revealed encephalitis and myelitis, and laboratory tests indicated inflammatory changes and oligoclonal bands in the CSF. However, excessive daytime sleepiness in patients with GFAP-A is an infrequent symptom. This contrasts with the findings reported by Fang et al. (1), who documented a case with GFAP-A who presented with subacute onset lethargy and T2 hyperintensities in the periventricular white matter. However, further details were not provided.

Excessive daytime sleepiness can stem from a range of factors, including sleep deprivation, sleep disorders such as OSA, restless legs syndrome, circadian rhythm disorders, and central hypersomnolence. Medical and psychiatric conditions such as depression, as well as medication-induced effects, may also be responsible (21). In this case, the patient had no previous medical conditions, medication history, or reported sleep disorders. Meanwhile, he experienced an acute worsening of excessive daytime sleepiness. As a result, we concluded that the patient’s excessive daytime sleepiness was due to severe SAHS and NT1, secondary to GFAP-A. The patient did not experience fluctuating drowsiness during the hormone tapering. Therefore, it is believed that the improvement in somnolence was due to the immunotherapeutic effect of methylprednisolone on GFAP-A rather than to the wakefulness-promoting effect of methylprednisolone.

Hypocretin is secreted by hypocretin-producing neurons in the hypothalamus, and its decline can present with narcolepsy-like symptoms, such as excessive daytime sleepiness, catalepsy, and rapid eye movement sleep behavior disorder (22, 23). Notably, our report marks the first documentation of decreased hypocretin in a patient with GFAP-A. However, the mechanism underlying the decrease in hypocretin in patients with GFAP-A is not well understood. Feneberg et al. (24) discovered that only patients with narcolepsy who experienced decreased hypocretin levels displayed increased GFAP levels in the CSF. Thus, it is believed that heightened GFAP is linked to gliosis of hypocretin-producing neurons and predicts more severe cellular damage. Additionally, it has been hypothesized that a decrease in hypocretin levels may be related to immunological factors secondary to the destruction of hypocretin neurons by GFAP peptide-specific CD8^+^ T lymphocytes. In the case of anti-Ma2-associated diencephalitis with NT1, immunohistochemical staining of brain sections confirmed that the CD8^+^ T cell-mediated autoimmune response induced by the anti-MA2 antibody caused the destruction of hypocretin-producing neurons (25), and a similar pathophysiological mechanism may be involved in our case. Notably, previous instances of various autoimmune antibodies, including anti-Ma antibodies (25–30), anti-Hu antibodies (31), AQP4 antibodies (32, 33) and anti-NMDA receptor antibodies (34) have been associated with decreased hypocretin, indicating a common yet unidentified antigen that attacked by multiple autoimmune antibodies in hypocretin-producing neurons. HLA DQB1*06:02, the main genetic factor responsible for susceptibility to NT1 (35), may also play a role in the development of an autoimmune response, ultimately resulting in the onset of secondary NT1 in the patient.

The substantial improvement in excessive daytime sleepiness and elevation of hypocretin levels observed in patients following hormone therapy implies that autoimmune neuronal damage has the potential for reversal at an early stage. Notably, our patient also presented with central respiratory events, including those that may have been associated with abnormal brainstem lesions, specifically in the pons. These lesions correspond to the functional brain network underlying the pathophysiology of central sleep apnea (36). Additionally, the concurrent disappearance of both central respiratory events and lesions supports this correlation. This suggests the importance of conducting screening and initiating treatment for central respiratory events in patients with GFAP-A who exhibit brainstem lesions.

GFAP-A is responsive to corticosteroid therapy but typically exhibits a relapsing-remitting course. Thus, in severe cases, additional treatments such as immunosuppressants may be necessary. While the patient in this case showed a positive response to corticosteroids, the persistence of NT1 warrants additional follow-up to monitor any potential regression in the patient’s condition.

## 4 Conclusion

In conclusion, we present the inaugural case of GFAP-A characterized by excessive daytime sleepiness as the primary manifestation, accompanied by a decrease in hypocretin and SAHA. The precise mechanism of this presentation remains elusive, but it is speculated to be associated with autoimmunity mediated by GFAP peptide-specific CD8+ T lymphocytes, with steroid treatment proving to be effective. This unique case highlights the diverse clinical spectrum of GFAP-A and the need for further research to unravel its intricate pathophysiological mechanisms.

## Data availability statement

The datasets presented in this article are not readily available because this is a case report. Requests should be directed to the corresponding author.

## Ethics statement

Written informed consent was obtained from the individual(s) for the publication of any potentially identifiable images or data included in this article.

## Author contributions

MT: Writing – original draft. SH: Writing – original draft. WG: Writing – review & editing. JZ: Writing – review & editing. ZH: Writing – review & editing. WL: Writing – review & editing. QS: Writing – review & editing. ZW: Writing – review & editing.
