# Supplementary figures and images for: Case report: Excessive daytime sleepiness as a presenting manifestation of autoimmune glial fibrillary acidic protein astrocytopathy
Source: Front Immunol. 2023 Dec 20;14:1302514. doi: 10.3389/fimmu.2023.1302514 (PMC10761546; doi:10.3389/fimmu.2023.1302514)

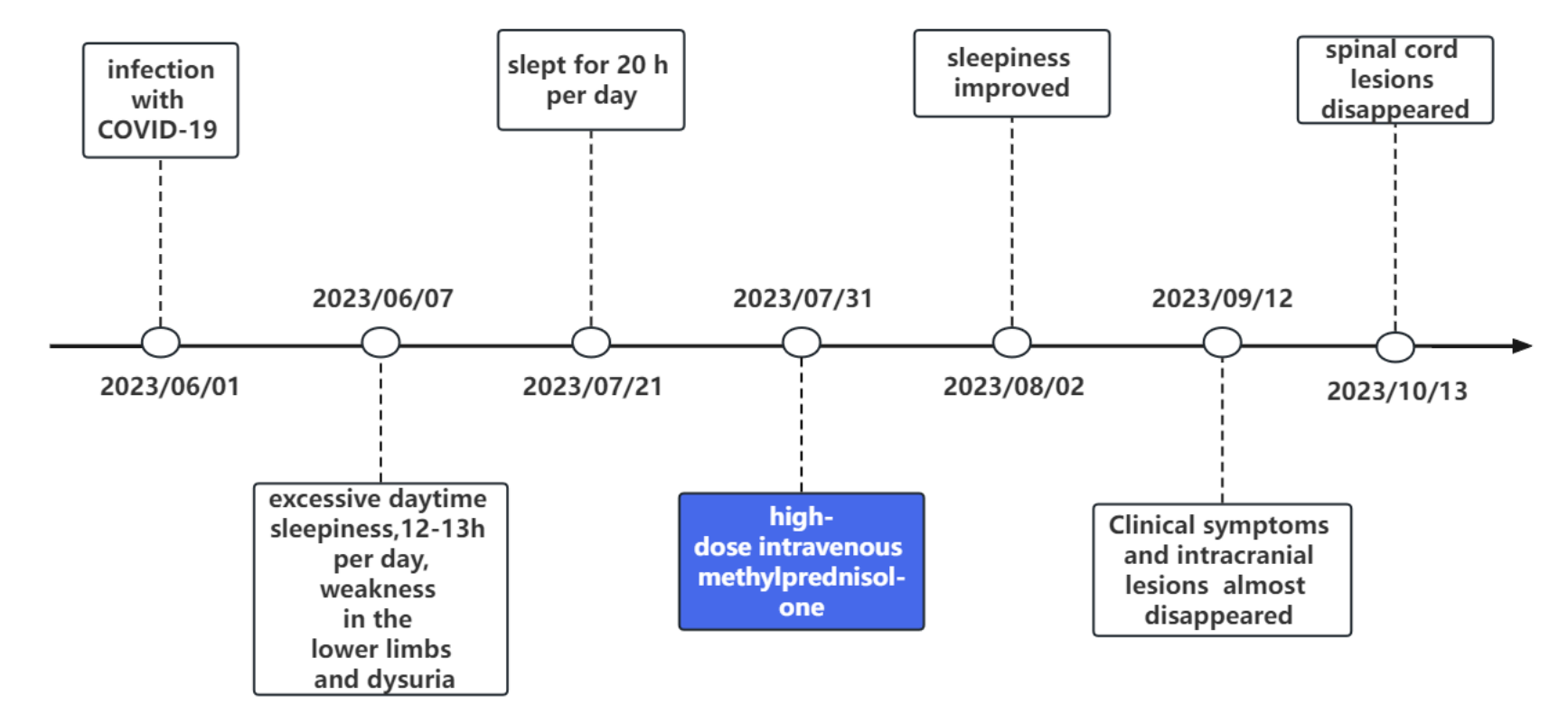

Supplement: Supplementary file 1 [file Image_1.tif]
