# Supplementary material for: Case report: Excessive daytime sleepiness as a presenting manifestation of autoimmune glial fibrillary acidic protein astrocytopathy
Source: Front Immunol. 2023 Dec 20;14:1302514. doi: 10.3389/fimmu.2023.1302514 (PMC10761546; doi:10.3389/fimmu.2023.1302514)
Supplement: Supplementary file 2 [file DataSheet_1.pdf]

CARE Checklist of information to include when writing a case report

CARE case report guidelines

(cc) BY-NC-ND

Topic	Item	Checklist item description	Reported on Line
Title	1	The diagnosis or intervention of primary focus followed by the words “case report”	Title
Key Words	2	2 to 5 key words that identify diagnoses or interventions in this case report, including "case report"	Keywords
Abstract (no references)	3a	Introduction: What is unique about this case and what does it add to the scientific literature?	Abstract
	3b	Main symptoms and/or important clinical findings	Abstract
	3c	The main diagnoses, therapeutic interventions, and outcomes	Abstract
	3d	Conclusion—What is the main “take-away” lesson(s) from this case?	Abstract
Introduction	4	One or two paragraphs summarizing why this case is unique (**may include references**)	Introduction paragraph 3
Patient Information	5a	De-identified patient specific information.	Case description paragraph1
	5b	Primary concerns and symptoms of the patient.	Case description paragraph1
	5c	Medical, family, and psycho-social history including relevant genetic information	Case description paragraph1
	5d	Relevant past interventions with outcomes	Not applicable
Clinical Findings	6	Describe significant physical examination (PE) and important clinical findings.	Case description paragraph1
Timeline	7	Historical and current information from this episode of care organized as a timeline	Supplementary material
Diagnostic Assessment	8a	Diagnostic testing (such as PE, laboratory testing, imaging, surveys).	Case description paragraph 2, 3 and 4
	8b	Diagnostic challenges (such as access to testing, financial, or cultural)	Case description paragraph 3
	8c	Diagnosis (including other diagnoses considered)	Case description paragraph4
	8d	Prognosis (such as staging in oncology) where applicable	Not applicable
Therapeutic Intervention	9a	Types of therapeutic intervention (such as pharmacologic, surgical, preventive, self-care)	Case description paragraph 4
	9b	Administration of therapeutic intervention (such as dosage, strength, duration)	Case description paragraph 4
	9c	Changes in therapeutic intervention (with rationale)	Not applicable
Follow-up and Outcomes	10a	Clinician and patient-assessed outcomes (if available)	Case description paragraph 4
	10b	Important follow-up diagnostic and other test results	Case description paragraph 4
	10c	Intervention adherence and tolerability (How was this assessed?)	Not applicable
	10d	Adverse and unanticipated events	Not applicable
Discussion	11a	A scientific discussion of the strengths AND limitations associated with this case report	Discussion paragraph 5,7and9
	11b	Discussion of the relevant medical literature **with references**.	Discussion paragraph 1,2,3 and 4
	11c	The scientific rationale for any conclusions (including assessment of possible causes)	Discussion paragraph 7and 8
	11d	The primary “take-away” lessons of this case report (without references) in a one paragraph conclusion	Conclusion
Patient Perspective	12	The patient should share their perspective in one to two paragraphs on the treatment(s) they received	Not applicable
Informed Consent	13	Did the patient give informed consent? Please provide if requested	Yes ☑ No ☐
